# Explainable artificial intelligence for mental health through transparency and interpretability for understandability

**DOI:** 10.1038/s41746-023-00751-9

**Published:** 2023-01-18

**Authors:** Dan W. Joyce, Andrey Kormilitzin, Katharine A. Smith, Andrea Cipriani

**Affiliations:** 1 University of Oxford, Department of Psychiatry, Warneford Hospital, Oxford, OX3 7JX UK; 2 Institute of Population Health, Department of Primary Care and Mental Health, University of Liverpool, Liverpool, L69 3GF UK; 3 Oxford Precision Psychiatry Lab, NIHR Oxford Health Biomedical Research Centre, Warneford Hospital, Oxford, OX3 7JX UK; 4 Oxford Health NHS Foundation Trust, Warneford Hospital, Oxford, OX3 7JX UK

**Keywords:** Health care, Computational biology and bioinformatics

## Abstract

The literature on artificial intelligence (AI) or machine learning (ML) in mental health and psychiatry lacks consensus on what “explainability” means. In the more general XAI (eXplainable AI) literature, there has been some convergence on explainability meaning model-agnostic techniques that augment a complex model (with internal mechanics intractable for human understanding) with a simpler model argued to deliver results that humans can comprehend. Given the differing usage and intended meaning of the term “explainability” in AI and ML, we propose instead to approximate model/algorithm explainability by understandability defined as a function of transparency and interpretability. These concepts are easier to articulate, to “ground” in our understanding of how algorithms and models operate and are used more consistently in the literature. We describe the TIFU (Transparency and Interpretability For Understandability) framework and examine how this applies to the landscape of AI/ML in mental health research. We argue that the need for understandablity is heightened in psychiatry because data describing the syndromes, outcomes, disorders and signs/symptoms possess probabilistic relationships to each other—as do the tentative aetiologies and multifactorial social- and psychological-determinants of disorders. If we develop and deploy AI/ML models, ensuring human understandability of the inputs, processes and outputs of these models is essential to develop trustworthy systems fit for deployment.

## Introduction

In this review article, we examine explainable AI ("XAI”) in the specific context of psychiatric/mental health applications. Across healthcare, there is an emerging skepticism for the ambitions of general XAI, with recommendations^1,2^ to avoid so-called “black box” models altogether. An AI is opaque or “black-box” when the computational mechanisms intervening between an input and the AI’s output are too complex to afford a prima facie description of why the model delivered that output—the exemplar case being deep neural networks where computational complexity affords remarkable flexibility usually at the cost of increasing opacity. Historically, inductive data-driven methods were considered difficult for humans to understand and this was recognised in early applications of AI in medicine^3^ where research favoured the explicit capture of clinical heuristics using symbolic propositions and inference mechanisms imported from formal logic. Similarly, when developing MYCIN^4^ the authors preferred decision trees because “in order to be accepted by physicians [the system] should be able to explain how and why a particular conclusion has been derived”. In mental health, the need for explainability was articulated in early AI-based diagnostic applications; for example, in developing DIAGNO-II^5^ statistical methods (linear discriminant functions and Bayesian classification) were compared to decision trees. In the absence of any clear performance advantages between the three methods, the authors concluded that decision trees were preferred because the data, the system’s structure and the computations performed all stood in close correspondence with clinicians’ domain knowledge alongside an assumption that clinicians use a similar sequential rule-in/rule-out style of reasoning when making diagnoses.

These examples center the structure and functioning of algorithms and suggest both should stand in close correspondence with a putative model of how clinicians reason with information about patients. Here, model structure refers to the model’s parameterisation whereas function refers to the computational processes that transform inputs to outputs (a concrete and tutorial example is given in Supplementary Information). In the contemporary literature, this is described as “intrinsic interpretability”^2^. As most contemporary AI methods used in healthcare applications are inductive, data-driven and very often, “black-box” (particularly given the popularity of deep learning methods) intrinsic interpretability’s prescription for a human-understandable correspondence between inputs and outputs of the black-box model are absent resulting in the development of post-hoc techniques where another algorithm operates in parallel to the ‘main’ black-box model to provide “explanations”.

**Fig. 1:**
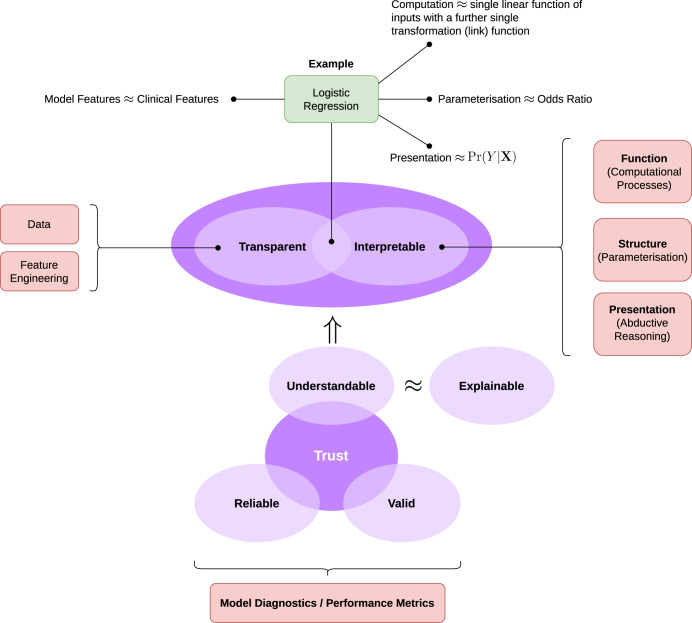
The transparency and interpretability for understandable models (TIFU). The TIFU framework operationalises “explainability” by focusing on how a model can be made understandable (to a user) as a function of transparency and interpretability (both definitions are elaborated in the main text). Algorithms and models will differentially satisfy these requirements and we show the example of logistic regression (in green, at the top of the diagram) as exemplifying a transparent and interpretable model.

The fundamental reason for pursuing explainability is that healthcare professionals and patients must be able to trust AI tools; more precisely, a trustworthy AI implies that human actors may rely^6^ on the tool to the extent they can economise on human oversight, monitoring and verification of the system’s outputs. To trust a deployed algorithm or model, we must have some understanding of how it arrives at an output, given some input. We therefore propose a framework for transparent and interpretable AI (Fig. 1), motivated by the principle of trustworthiness^7^ using the following rubric:

**Definition** (Understandable AI). For an AI to be trustworthy, it must be valid, reliable and understandable. To be understandable, an AI must be transparent and interpretable and this is an operationalised approximation for explainability.

In what follows, we present the TIFU framework (Transparency and Interpretability For Understandability) focusing on understandability as a composite of transparency and interpretability. The important concepts of model reliability and validity are beyond the scope of this work but have received attention and are well described in the literature^8^; briefly, to be reliable and valid, a model’s predictions or outputs must be calibrated and discriminating with respect to an observed outcome (or ground truth) and in addition, be generalisable (i.e. externally validated) so the model remains accurate and useful when deployed on new data not used during the model’s development^9–11^.

We proceed by first, surveying the mental health and psychiatric literature claiming to deliver explainable AI in a variety of application domains. Then, we highlight the connections to existing literature, drawing together consistent and concrete definitions that support the TIFU model. Throughout, we adhere to a convention of discussing understandability rather than “explainability”. Finally, we conclude with observations and recommendations for building systems that adhere to TIFU.

## Diverse definitions

To motivate our proposal, we searched PubMed and established that specific applications of XAI in mental health and psychiatry first began appearing in 2018 shortly after the inaugural International Joint Conference on Artificial Intelligence Workshop on Explainable AI in 2017^12^. We then surveyed papers published from 1st January 2018 through 12th April 2022 to examine how the term “explainable” is used in this literature. We found a diversity of definitions with a loose—often vernacular—meaning. We located 25 papers eligible for review, of which 15 were original research and 10 were reviews (see Supplementary Information for search details).

In Table 1, we summarise the 15 original research articles, grouped by the application (predominantly, in neuroimaging and survey data). Notably, in neuroimaging applications, where deep learning methods were most used, we found that the definition of explainability almost always defers to the XAI method or technique used (which were most often feature importance methods e.g. Shapley^13^ and LIME^14^). Occasionally^15,16^, methods with explainability or interpretability “by design”^2^ were used; these papers both used regression-based methods. Further, only three papers^17–19^ evaluated their proposed explainable AI with respect to how humans might make use of the explanations—arguably, an essential ground-truth for a successful XAI implementations. The situation was notably different in studies making use of survey data where evaluation of how humans might understand the AI’s inferences, discoveries or predictions were more common as were studies that attempted a more explicit definition of what they intended “explainability” to mean and the same studies were less likely to simply defer to the methods used.

**Table 1:** Original research reporting applications of explainable AI in mental health.

Citation	Application	Methods	XAI methods	Define	Evaluate	Defers to method
*Neuroimaging/EEG*						
Chang et al., 2020^46^	P, D	DL	FI	No	No	Yes
Supekar et al., 2022a^17^	P, D	DL	FI	No	Partial	Yes
Supekar et al., 2022b^18^	P, D	DL	FI	No	Partial	Yes
Kalmady et al., 2021^15^	P, D	DL, ER	Des, FI	Yes	No	Yes
Bučková et al., 2020^16^	P	R	Des	Partial	No	Yes
Ben-Zion et al., 2022^47^	D	R	FI	No	No	Yes
Al Zoubi et al., 2021^19^	P	DL, R, SVM, RF, NB, XG	FI	No	Partial	Yes
Smucny et al., 2021^48^	P	DL, MLP, SVM, RF, NB, K*, DT, AB	FI	No	No	Yes
*Survey*						
Mishra et al., 2021^49^	P, D	DT	FI, CI	Yes	Yes	No
Ammar et al., 2020^29^	DM	KG	Pr	Yes	Yes	No
Schaik et al., 2019^50^	P, D	R, DR	Des	Partial	No	No
Jha et al., 2021^31^	P, D	R, SVM, RF, NB, BN	Des, Pr	No	Yes	Yes
Byeon, 2021^20^	P, D	SVM, RF, XG, AB, LGB	Pr, FI	Partial	Yes	Yes
Ntakolia et al., 2022^43^	P	R, SVM, RF, XG, kNN, DT, MLP	FI	No	Yes	No
*Physiological*						
Jaber et al., 2022^30^	P	RF	Pr, FI	Yes	Yes	No

Application: *P* prediction, D discovery, *DM* decision making; Methods: *DL* deep learning, *ER* ensemble of regressions, *R* regression, *SVM* support vector machine, *RF* random forest, *NB* Naive Bayes, *XG* XGBoost, *MLP* multilayer perceptron, K* K-star instance-based classifier, *DT* decision trees, AB AdaBoost, *KG* knowledge graph, *LGB* light gradient boost, *kNN* k-nearest neighbour; XAI Methods: *FI* feature importance, *Des* by design, *CI* causal inference, *Pr* presentation.

The uses of AI were most often a combination of prediction and discovery (8 of the 15 studies); by this, we mean that while e.g. classifiers were built to discriminate between patients and controls (with the intent of making predictions for new patients), often, the trained models were then dissected to provide insights on the high-dimensional multivariate input data—similarly to how inferential methods are used in classical statistical analyses. This may signal that when researchers are faced with multivariate data but an absence of clear a priori knowledge about the application that would assist engineering a solution, the flexibility of supervised learning delivers automated feature selection. It is no surprise that this approach was prevalent in neuroimaging studies, where the use of deep learning (especially, image processing architectures) is notable for studies which report a combination of prediction and discovery.

Finally, we note that when an array of ML methods were used (e.g. testing and then selecting a best-performing classifier in either neuroimaging or survey data), with one exception^20^, there was no definition of what explainability meant and the authors deferred to the XAI method used. Thematically, almost all original research papers follow a pattern of describing why XAI is important, usually presenting a prima facie argument (e.g. that human operators need to be able to understand what the AI is delivering) with few explicitly addressing a definition with respect to the application domain or methods used. More often, rather than being explicitly defined—or addressing how the research delivers explainability—papers defer to methods (most commonly, feature importance) or assume XAI is conventional wisdom.

## A framework for understandable AI/ML in mental health applications

Given the diversity of definitions of “explainability” we now describe a framework for “understandable AI/ML” for mental health research that centers transparency and interpretability—both concepts which possess more consistent meanings in the literature—and recalling our earlier definition, we propose understandability as the most concrete approximation^21^ to the multifarious definitions and uses of the term “explainability”. To do this, we anchor our definitions to models that have intrinsic interpretability or are understandable by design (i.e. linear statistical models). A tutorial example (comparing a fully understandable linear model to an opaque neural network model) is given in Supplementary Information.

In Fig. 1, we show the TIFU framework. An AI/ML algorithm takes some input and performs operations to derive a feature space which is the basis for downstream computations that implement the desired functionality e.g. classification, regression, function approximation, etc. The derived feature space is usually optimised to ensure the downstream task is tractable. If we denote the output of a model *y*, the multivariate input **x** and *f*(**x**) being some function mapping from inputs to the feature space (which may be a composition of many functions) and *g*(*f*(**x**)) be the downstream process (that operates on the feature space and may also be a non-trivial composition of functions) then:

**Definition** (Transparency). The inputs **x** and feature space *f*(**x**) of a model should either

- stand in close correspondence to how humans understand the same inputs or,
- relationships between inputs (and their representation in features space) should afford an interpretation that is clinically meaningful

For example, if the inputs to a model are *Age* and performance on some cognitive task, *TestScore*, then the model is feature transparent if:

- trivially, the feature space is identical to the inputs, *f*(**x**) ≡ **x**,
- the feature space can be described e.g. as an explicit function of the inputs *f*(**x**) = *Age* + *TestScore*^2^—in this example, the function may represent feature engineering that includes human-expert domain knowledge relevant to the application,
- the feature space can be represented to a human operator such that differences and similarities between exemplars (i.e. two different patients) preserves or affords a clinical meaning e.g. exemplars with similar *TestScore* values aggregate together even if they differ in *Age*. In this example, let *f*(**x**_1_) represent an individual with a low *TestScore* and younger *Age* (a clinical group representing premature onset of a cognitive disorder) and *f*(**x**_2_) represent an individual with a low *TestScore* and higher *Age* (representing another clinical group)—if *f*(⋅) is a non-trivial function, we need to provide a mechanism that exposes why **x**_1_ and **x**_2_ are represented differently/similarly under *f*(⋅) consistent with a human expert’s differentiation of the two cases; an obvious method being distance-preserving mappings for unsupervised algorithms or conformity measures for e.g. supervised classification with deep learning^22^.

We need not commit to any one way of defining “relationships” between inputs—they could be probabilistic (different exemplars have similar probabilities of membership to components of a mixture model of the feature space), geometric (distances on some manifold representation) or topological (such as nearest-neighbour sets). It matters only that the feature space is represented in a way that aligns with the clinical problem/population (see for example, Supplementary Information Figure 2).

**Definition** (Intepretable). For a model to be interpretable—akin to the concept of algorithmic transparency^21^—we require one or more of the following:

- The function (computational processes) of *g*(⋅) can be articulated so the outputs can be understood as transformations of the inputs.
- The structure (parameterisation) of *g*(⋅) can be described and affords a clinical interpretation.
- The presentation of *g*(⋅) allows for a human operator to explore qualitative relationships between inputs and outputs (i.e. the behaviour of the model).

Clearly, criteria (a) will be difficult to achieve in all but the simplest cases (e.g. primitive arithmetic operations) and similarly, criteria (b) will be difficult to achieve for methods lacking the theoretical underpinning of e.g. linear statistical models. Consequently, criteria (c) is likely to be leveraged in many applications where *g*(⋅) is some non-trivial function of it’s inputs.

For example, logistic regression admits all three of the desiderata for interpretability as follows:

- The computational processes (function) are: first compute a weighted sum of the inputs *f*(**x**) = **x**^⊺^***β*** e.g. representing the log odds of **x** being a positive case on the logit scale; then compute a “link” function that converts the unbounded weighted sum into a probability $g(f({{{\bf{x}}}}))=1/(1+\exp (-f({{{\bf{x}}}})))$.
- The parameterisation (structure) ***β*** affords a direct interpretation as odds ratios for each of the inputs *x*_*i*_ ∈ **x** with respect to the output.
- The presentation is straight-forwardly that Pr(*y* = 1∣**x**) = *g*(*f*(**x**))—although we might consider a format established to be more compatible with clinician reasoning, e.g. natural frequencies instead of probability statements^23,24^.

The obvious stress-test for our definition of transparency and interpretability (to deliver an understandable model) are applications of deep learning. For example, in ref. ^15^, the authors use convolutional networks to pre-process resting state fMRI data and then downstream, classify cases into those likely to have obsessive compulsive disorder. Their modelling uses three different architectures; two that operate on the fMRI data directly (where *f*(⋅) is composed of two layers of convolution, followed by max pooling and a linear output layer that implements supervised feature selection) and another where previously engineered, anatomically-parcellated classifiers provide a feature representation. The two architectures that make use of convolutional layers (at least, as they are presented^15^) do not meet the criteria for transparency or intepretability. However, the third model (parcellation-based features) does meet the transparency criteria because for an individual patient, each anatomical-parcellation classifier delivers a feature value proportional to that brain region’s probability of being ‘pathological’ (e.g. being similar or different to the prototype for a disorder or healthy patient). Further, for the interpretability criteria, we conclude that as presented, although the upstream parcellation system meets criteria (a) and (b) overall, the results as presented in ref. ^15^ marry with the presentation criteria (c).

## Presentation and clinical reasoning

In our definition of understandability (as transparency and interpretability) we rely heavily on a human operator being able to relate the behaviour of algorithms (and their inputs) to their everyday professional expertise. Our inclusion of “presentation” as a third component of interpretability is because we expect a model’s operation will be too complex. To this end, consistent with others^21^, we add that the model must present input/output relationships aligned with the cognitive strategies that clinicians use. We focus on abductive and inductive (in contrast to deductive) inference as the most applicable framework^25–28^. Some of the literature surveyed leverages user interface design to present the outputs of complex models a way familiar to clinicians^16,20,29–31^. Even for generalised linear models, clinicians may struggle to directly interrogate the structure (parameterisation) and function (computations) but of course, have recourse to interpretability afforded by the structure of these models (criteria b).

Interactive visualisation may allow clinicians to “probe” the model for changes in the probability of an outcome (e.g. a diagnosis) by manipulating the input features (such as presence/absence of symptoms) and this assists users to develop a qualitative understanding of the relationship between inputs/outputs. By analogy, nomograms^32^ allow a user to visually compute the output of complex mathematical functions without access to explicit knowledge of the required operations (i.e. the function/computational processes). In the deep learning literature^33^, a similar idea estimates the change in a classifier’s output i.e, the change in *g*(*f*(**x**))) for systematic changes in the input (**x**)^34^ and similar perturbation techniques^35^ can be applied to components of models (i.e. to *f* and *g* seperately, or if *g* is a non-trivial composition of functions). However, the focus of these predominantly engineering solutions is on image processing systems and there is a dearth of literature that explores the specific context of clinical reasoning e.g. how an AI might assist with diagnosis outside of the narrower but familiar domain of imaging.

**Fig. 2:**
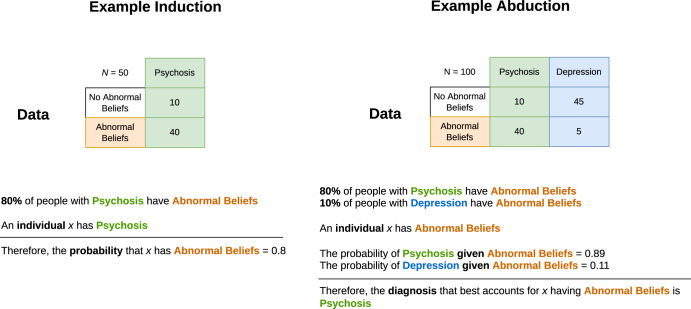
Examples of inductive and abductive inference. Using the example of making diagnoses, the left panel shows an inductive inference using a statistical syllogism—a probable conclusion for an individual (in the example given, the probability an individual will experience abnormal belief symptoms, given they have a diagnosis of a psychotic disorder) is obtained by generalising from available data (i.e. the contingency table showing the proportions of patients with psychosis who have abnormal beliefs). In the right panel, abductive inference affords computing the best or most plausible explanation (i.e. a diagnosis of psychosis or depression) for a given observation (that a person has abnormal beliefs) using the available data (a contingency table for two diagnoses).

The requirements for presentation will need alignment with the different use-cases for AI. With respect to the “Application” column of Table 1: for discovery applications of AI, inductive reasoning allows us to use statistical or probabilistic information to generalise from examples; in Fig. 2, if we know that 80% of people with psychosis (an hypothesis or diagnosis, *D*) have abnormal beliefs (evidence, or signs/symptoms *S*) then induction allows us to make generalisations about individuals with *D* given what we know about the relationship with *S*, or symbolically, *D* → *S*. Presentation as induction would be useful when dissecting disorder sub-types^36^ and neuroscientific discovery^37^ where dimensionality reduction and unsupervised clustering methods would align with an inductive presentation.

More relevant to decision making and prediction applications is abductive reasoning; the process of inferring which hypotheses are best supported given some evidence—for example, in diagnostic reasoning, we can consider evidence (as signs/symptoms, *S*) and hypotheses as a number of candidate diagnoses *D*_1_, *D*_2_, …*D*_*n*_. We aim to infer which *D*_*i*_ best accounts for the evidence *S* and this is compatible with conditional probability and Bayes theorem; that is, we seek Pr(*D*_*i*_∣*S*) ∝ Pr(*S*∣*D*_*i*_) ⋅ Pr(*D*_*i*_). In contrast to induction, we are inferring *S* → *D*. Inductive inference differs from deductive inference because although the “direction” is *D* → *S* in deductive inference, the truth of *D* and *S* are absolute; for example, in Fig. 2, deductive inference would assert that it is necessarily true that if a person has psychosis, they definitely have abnormal beliefs (cf. the probabilistic interpretation afforded by inductive reasoning). To re-use the example of making a diagnosis, it is clear that psychiatric diagnoses have “many-to-many” mappings with the underlying biology^38–40^, the probabilistic nature of psychiatric diagnosis (i.e. the mapping of signs/symptoms to diagnoses) has long been recognised^41^ and consequently, influenced the dimensional characterisation of disorders^42^. Here, we suggest that an abductive presentation would be most suitable.

## Conclusion and recommendations

We now describe the implications of both our survey of the literature on XAI in mental health and the proposed TIFU framework. Firstly, we note that the application of XAI in mental health were broadly prediction, discovery or a combination of the two. Second, we require understandability because clinical applications are high-stakes. Third, we expect that when we deploy AI tools, they should assist clinicians and not introduce further complexity. In our review of recent literature on the topic of explainable AI in mental health applications, we note that in 8 of 15 original research papers, prediction and discovery are considered together—using our framework, this would require separate consideration of the TIFU components with respect to each task (prediction and discovery) separately.

Our first recommendation is driven by the diversity of AI/ML techniques deployed in applications on clinical survey data e.g., refs. ^20,31,43^—which here, means voluminous, tabulated data with high numbers of input (independent) variables and where there is no a priori data generating model or domain knowledge which enables human-expert feature selection/engineering. These applications were characterised by (a) the use of multiple AI/ML methods and their comparison to find the “best” model and (b) post hoc interrogation of the model (e.g. by feature importance methods) to provide a parsimonious summary of those features for the best performing model. The AI/ML methods are essentially being used to automate the exploration of data for signals that associate with an outcome of interest while simultaneously, delivering a functioning classifier that could be deployed to assist clinicians.

- Recommendation One: When multiple AI/ML techniques (that are not transparent and interpretable) are used to discover which inputs are features reliably associated with an output of interest, the “discovered” feature/output associations should be then be tested by post hoc constructing a transparent and interpretable model which uses only those discovered features.

In essence, we are suggesting that the wholesale application of AI/ML methods should be seen as exploratory analyses, to be followed by constructing a transparent and interpretable model. For example, in ref. ^43^ the best performing classification method was shown to be XGBoost and post hoc analyses with SHAP values identifies a subset of inputs which have most utility as features for classifying whether an individual was likely to experience a change in mood state during the Covid pandemic lockdown. Our recommendation would mean constructing a model with the discovered features—clearly identifying the mapping from inputs to features—examining it’s performance and ensuring that the criteria for interpretability are met. A counter argument would be that this leads to circular analyses—or “double dipping” the data—leading to sampling bias in the interpretable model. This may be true, but it is a limitation of the approach in general because if the discovered features migrated into the interpretable model are robust, the understandable model should still perform when prospectively evaluated in another validation sample. This latter step then ensures that the model is valid, reliable and understandable which can only provide reassurance to clinicians using the system when it is deployed. This recommendation is similar to “knowledge distillation”^44^ and methods have developed wholesale for extracting decision trees from deep learning models^45^.

Our next recommendation is driven by the observation (see Table 1) that deep multi-layer networks were used to implement classification as a downstream task and, essentially, supervised learning of a feature space representation for very-high dimensional inputs. In these cases, we can identify a partition between the upstream component that performs feature representation *f*(⋅) and the downstream task *g*(⋅).

- Recommendation Two: When using high-volume, high-dimensional (multivariate) data, without a priori domain-specific constraints and instead, we wish to automate reducing the data to feature representations *f* essential for a downstream task *g*: when the methods used to implement *f* are neither transparent (data, features) or admit interpretability (function, structure) they should be engineered and then deployed as a separate component for use in interpretable methods for the downstream task *g*.

Essentially, we are recommending that when we rely on opaque models for processing high-volume/dimensional data, they should be treated as a pre-processing ‘module’ and the downstream task *g* that depends on the feature representation should be implemented using models that meet interpretability criteria. As an example, the anatomical parcellation model developed for identifying people with OCD^15^ exemplifies this. Our recommendation would be that instead of using subsequent multiple layer networks for classifying OCD, a simpler interpretable model should be preferred because; then, the outputs of each anatomically-parcellated pre-processing ‘modules’ are transparent and the downstream classification task would be interpretable.

A counter argument might be that the upstream feature representation might not be compact enough, or, that the multiple layers in the downstream classifier are required to flexibly aggregate the feature representation for *g*; if this is the case, then the model will necessarily remain opaque, lack understandability and may well be vulnerable to over-fitting both *f* and *g* and therefore, unlikely to be useful in high-stakes applications.

We have consistently described AI/ML models as being composed of an “upstream” component that delivers a feature representation, *f*(⋅), coupled to another “downstream” process, *g*(⋅), that uses the feature representations to perform e.g. prediction, discrimination/classification and so on. This may not be appropriate to all AI/ML methods—however, from our review of current XAI in mental health and psychiatry, this is how AI/ML methods are being used.

Our proposed TIFU framework simultaneously lowers our ambitions for what “explainability” is while emphasising and making concrete a definition that centers (a) computational processes and structures, (b) the presentation of outputs and (c) the way that data or inputs relate to the clinical domain. Our approach draws on principles in the general XAI literature—notably, ref. ^2^ and ref. ^21^—extending these principles to specific considerations for psychiatry and mental health including inductive and abductive inference and the differing nature of understandability for prediction, discovery and decision-making applications. To conclude, our ambition for the TIFU framework is to improve the consistency and specificity of what we mean when we allude to “explainability” in research involving AI and ML for mental health applications.

### Reporting summary

Further information on research design is available in the Nature Research Reporting Summary linked to this article.

## Supplementary information

Supplementary Material

Reporting Summary

## Data Availability

The simulated data presented is freely available at https://github.com/danwjoyce/explain-simple-models.

## Code availability

The simulation code presented is freely available at https://github.com/danwjoyce/explain-simple-models.
