# Supplementary material for: Explainable artificial intelligence for mental health through transparency and interpretability for understandability
Source: NPJ Digit Med. 2023 Jan 18;6:6. doi: 10.1038/s41746-023-00751-9 (PMC9849399; doi:10.1038/s41746-023-00751-9)
Supplement: Supplementary file 1 — Supplementary Material [file 41746_2023_751_MOESM1_ESM.pdf]

Explainable Artificial Intelligence for Mental Health through Transparency and Interpretability for Understandability (Supplementary Information)

Dan W Joyce[*,1,3], Andrey Kormilitzin[1], Katharine Smith[1] and Andrea Cipriani[1,2]

[1]University of Oxford, Department of Psychiatry, Warneford Hospital, Oxford, United Kingdom, OX3 7JX
[2]Oxford Precision Psychiatry Lab, NIHR Oxford Health Biomedical Research Centre, Warneford Hospital, Oxford, United Kingdom, OX3 7JX
[3]Institute of Population Health, Department of Primary Care and Mental Health, University of Liverpool, Liverpool, L69 3GF
[*]Corresponding author; email: danjoyce@liverpool.ac.uk

Search Method

For the literature reviewed in Table 1: Data were extracted from PubMed / MedLine using the search: `(explaina*) AND ("artificial intelligence" OR "machine learning") AND ("mental health" OR "psychiatry")` in the title and abstract fields.

The date range was 1st January 2018 through 12th April 2022 and extracted on the latter date. The search delivered 32 papers, of which 7 were excluded as they addressed applications in 1) surgical mortality 2) an editorial preface to a special issue 3) psychophysics of visual perception 4) inflammatory processes in osteoarthritis 5) polypharmacy (only tangentially linked to psychiatry) 6) quantifying altered states of consciouness and 7) feature set selection in osteoarthritis.

The full-text of the remaining 25 papers were reviewed and the 15 which presented original research retained.

Literature Summary

For papers reporting original research, we assessed the following properties:

- the broad domain addressed in the research: most studies were on survey or neuroimaging data, with one examining physiological data
- the intended application (i.e. AI for prediction/forecasting, discovery or decision making/decision support): finding that most studies contained a prediction and discovery component
- what AI/ML methods were used: in most survey-based papers, multiple methods were compared especially in applications where prediction performance was tested and in neuroimaging, deep learning methods dominated
- which XAI methods were used: we grouped these into feature importance, explainability "by design", causal inference and presentation/visualisation methods

finding that feature importance methods dominated across applications

- whether the research evaluated the claimed “explainability”: finding that in papers using survey data, evaluation was more commonly reported
- whether the research deferred “explainability” to the technical method used: finding that half of survey data-based applications deferred to the method; in papers that did not define explainability in their application, they always deferred to the method used (e.g. in all of the neuroimaging studies)

Of note, we did not rate the quality of definitions (i.e. whether or not they were detailed or precise) – rather, we examined if the paper made any attempt to define terms such as “explainability” beyond stating that it was merely important or necessary.

A Tutorial Example of Structure and Function

To motivate our discussion of structure and function in the TIFU framework, we introduce a simple toy example of recommending whether or not a patient should receive antidepressant medication based on seven predictor variables; the patient’s age, natal sex, history of previous major depressive episodes, previous treatment with medication and/or psychotherapy as well as the current episode severity and duration in weeks. We simulated 3759 patients where the decision to offer medication was a non-linear function of the seven predictor variables and divided this into a training and testing set of 2643 and 1116 samples respectively.

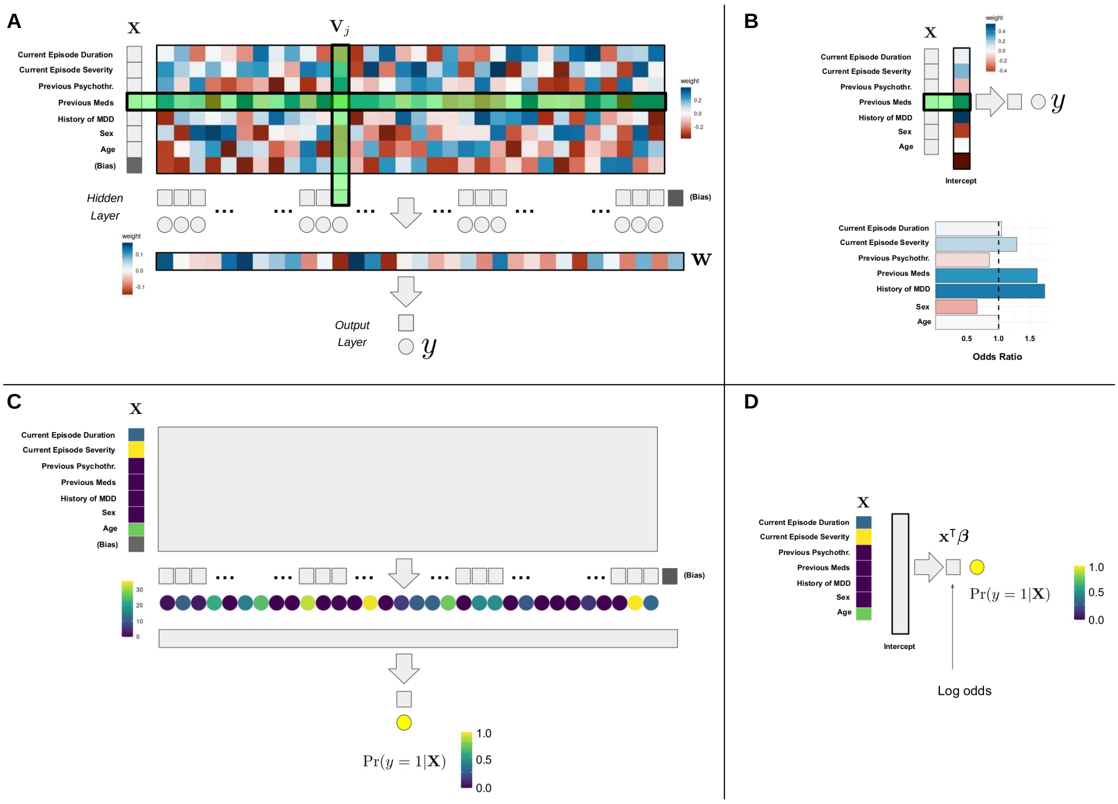

Supplementary Figure 1: Structure and Function in a Toy Model to Predict Antidepressant Prescribing from Clinical Characteristics: (A) The **structure** of a simple neural network consisting of a 7-node input layer fully (densely) connected to a 32-node hidden layer, fully connected to a single output node. Grey squares indicate computations over the preceeding layer's outputs; grey circles represent (usually non-linear) operations or activation functions. (B) the structure of an equivalent logistic regression model for the same problem presented using the same description of weights (parameters) and operations (weighted linear sums and activation functions). (C) The **function** of the neural network showing an example patient and the pattern of activations as computations proceed ("feedforward") from the input to output layer via the hidden layer. (D) The same example patient being "fed-forward" in the logistic regression model

In Supplementary Figure 1, we show the **structure** of a feedforward neural network model (panel A) with 7 inputs nodes, each corresponding directly to one of seven pre-

dictor variables. We perform no feature engineering on this data so that the inputs to the model directly correspond to the clinical variables used to make predictions. In this feed-forward network, the layer of input nodes therefore have *activations* identical to the values of the inputs (denoted by the column vector $\mathbf{x}$). This input layer is densely (fully) connected to 32 hidden nodes by a matrix of adjustable weights $\mathbf{V}$ (or parameters) shown as the rectangular red/blue coloured matrix; this is to enable the hidden layer to capture interactions or more precisely, representations of the input as linear weighted sums the input variables. The outputs of the hidden nodes are then transformed through another vector of weights $\mathbf{w}$ which transform the outputs of the hidden layer to a single output node y whose output is proportional to the probability of offering (or not) antidepressant medication ($y = 1$ or 0 respectively).

The **function** of the network can be visualised as a pattern of activations propogating through the network (panel C) as follows; each input node has activation identical to the values of the predictor (input) variables – shown as the coloured squares for an example patient at the left of panel C. This example patient has the following characteristics (corresponding to the visual representation in panels C and D): they are a 46 year old female, with no previous history of MDD, medication or psychotherapy treatment and with a MADRS score of 60 and episode duration of 20 weeks. The logistic regression model and the neural network recommend antidepressant medication with probability $\Pr(y = 1|\mathbf{x}) = 0.99$.

These activations feed-forward to the hidden layer where each node computes a net input (shown as grey squares) as a weighted sum $a_j = \mathbf{x}^{\mathsf{T}}\mathbf{V}_j$ where $\mathbf{V}_j$ is the column vector (e.g. the vertical green bar in panel A) of the weights connecting the hidden node j to every input node i. Conversely, this means the effect of an isolated input node i is "distributed" over *all* hidden nodes – illustrated by the horizontal green bar in panel A. Next, each hidden node computes it's activations by taking the net input a_j and delivering an output through a rectified linear activation (ReLU) function $f(a_j) =$

$\max(0, a_j)$ – represented in panel C as the row of coloured circles. ReLU hidden nodes effectively "switch off" hidden nodes where the net input a_j falls below a threshold which by convention, is modelled using a so-called bias node that can be viewed as similar to the intercept in a traditional linear regression model. Finally, the output layer has only a *single* node that, similarly, computes a linear weighted sum of inputs (i.e. the outputs of the hidden layer): $b = \mathbf{f}\mathbf{w}^{\intercal}$ (this operation is again shown as a grey square) where $\mathbf{f}$ is the row vector of 33 hidden-layer node outputs (32 hidden nodes plus a single bias node) and $\mathbf{w}$ is a row vector of weights connecting every hidden node to the output y. Instead of a ReLU function, however, the output node y computes a sigmoid (logistic) function of it's inputs resulting in $y = g(\mathbf{f}) = \frac{1}{1+\exp(-\mathbf{f})}$ which approximates the probability that a patient $(\mathbf{x})$ is recommended a prescription for antidepressant treatment. For completeness, the weights $\mathbf{V}$ and $\mathbf{w}$ were estimated using stochastic gradient descent. Note that the final output of the network, y, can be written compactly as a sequence of function compositions: $y = g(f(\mathbf{x}))$ or equivalently $y = g \circ f$, emphasising that the output depends on the input passing through one layer of computations (f) which feed into the second layer (g) to arrive at the output (y). The "deeper" the network, the more function compositions are involved.

Contrast with a logistic regression model shown in panel B; here, we have adopted the same diagrammatic convention of showing the structure as weights (commonly referred to as "betas" or the coefficients of the model) and computations (panel D) where the input x is multiplied by the weights $\boldsymbol{\beta}$ (grey square) and then transformed via a sigmoid or logistic function (grey circle) to arrive at an output proportional to the probability of recommending an antidepressant. In essence, logistic regression can be viewed as a trivially-simple neural network without hidden layers, where the input layer is densely and directly connected to the output node. The weights/parameters are estimated using an iteratively re-weighted least squares algorithm. The output of the logistic regression is a single function of the input: $y = g(\mathbf{x}) = \frac{1}{1+\exp(-\mathbf{x}^{\intercal}\boldsymbol{\beta})}$

Both models provide the same behaviour when seen as a "black box" – that is, modelling the probability of recommending an antidepressant *given* a patient's characteristics but they achieve this through differing model **structure** (architecture) and **functions** (computations). Relevant to the TIFU concept, the neural network allows for more flexible representations by virtue of the input activations being "distributed" over the hidden layer nodes. The logistic regression model can only account for weighted linear sums of the seven input variables – meaning that there are no modelled interactions between variables.

To **understand** how the neural network arrives at an output, we must recognise the output (y) results from two function compositions of multivariate inputs, neither of which have *prima-facie* **transparency** with respect to the input $\mathbf{X}$ or outputs y and $\Pr(y = 1|\mathbf{X})$.

Contrast with the logistic regression model (panel B) where the weights/parameters (**structure**) possess a well-developed formal relationship to the predictor variables i.e. exponentiating the weights yields the direct **interpretation** of each predictor variable having an associated odds ratio. Similarly, the function of the logistic regression model can be easily interpreted – the weighted sum $\mathbf{x}^{\intercal}\boldsymbol{\beta}$ (computed by the grey square in panel D) is the log odds or probability of $y = 1$ on the logit scale [1]. The computation performed over the weighted sum (the grey circle in panel B and C) is the logistic function which maps log odds to the probability scale resulting in $\Pr(y = 1|\mathbf{X})$. In the logistic regression model, we retain the inputs-to-model structure (odds ratios) and function (the effect on $\Pr(y = 1|\mathbf{X})$ of selectively modifying one or more inputs holding all others constant) offering interpretablility by design.

Supplementary Figure 2 shows that we can qualitatively visualise the outputs of the hidden layer $f(\cdot)$ in an attempt to provide interpretability of the function of the model

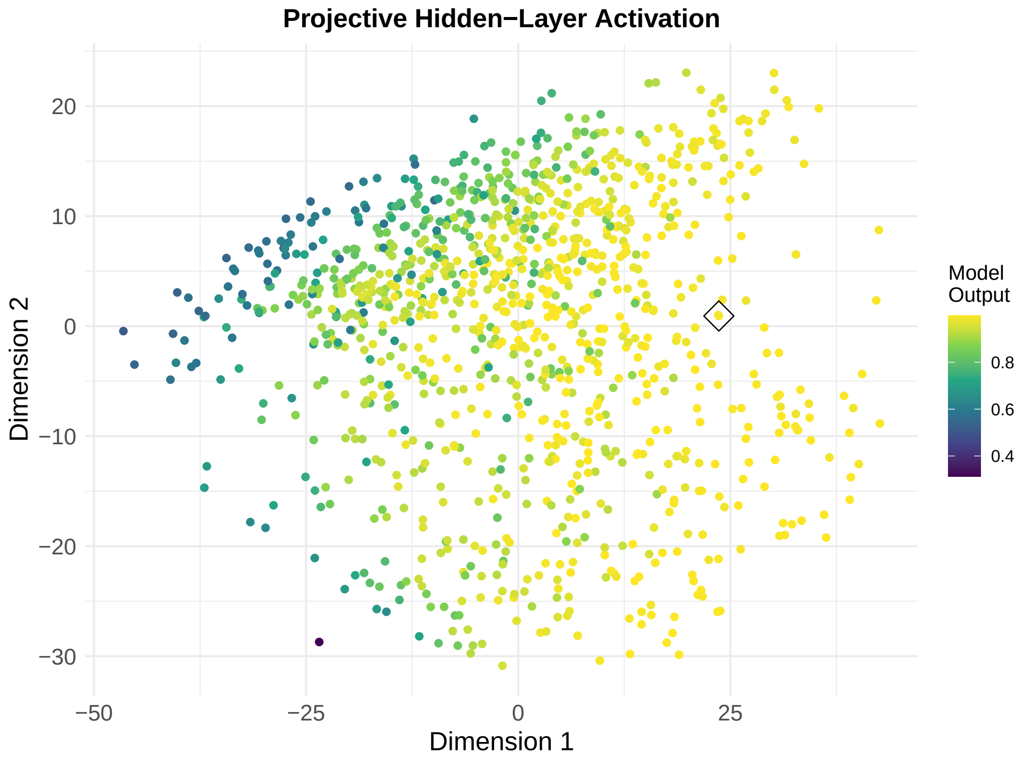

Supplementary Figure 2: Two-dimensional projection (by metric multidimensional scaling) of the activations of the 32 hidden layer nodes ($\mathbf{f}$) induced by each of 1116 patients, where the colour represents the model's output, $\Pr(y = 1|\mathbf{x})$. The black diamond shows the location corresponding to the example patient from Supplementary Figure 1

with respect to the outputs. We use metric multidimensional scaling [2] to "project" the native 32 dimensional space of activations into two, unitless dimensions that stand in correspondence to the inputs in a non-trivial way; for example, the patient from Supplementary Figure 1 is a 46 year old female with no treatment or depression history presenting with severe symptoms of duration 20 weeks and the location in this dimensionality-reduced space of activations is shown with a black diamond. There is no straight-forward way of directly mapping these input variables to the two-dimensional space of activations in a way that would be transparent or interpretable. Further, at least as we present here, the presentation of this information does not help us with ab-

ductive (or inductive) reasoning about why this recommendation was made. While the projection shows some qualitative pattern in relation to the probability of being recommended an antidepressant medication, it is far from clear how to *use* this to aid human interpretation.

In summary, the model becomes further removed from *prima facie* interpretability as a consequence of the structure (architecture of the model) and function (i.e. corresponding to the depth or number of function compositions). We should note two further points: i) that the example "toy" neural network presented is substantially less complex compared to a typical application of deep learning in contemporary AI/ML and ii) with some knowledge of linear algebra, we could describe a systematic relationship between the inputs and hidden-layer nodes' net inputs (structure and function), but this is complicated by there being a non-linear function $f(\cdot)$ and this is unlikely to be available to a clinician or patient using such a model.

Given this, we have *post-hoc* methods such as LIME [3][1] and Shapley-based methods [4] which both anchor the concept of "explainability" to perturbation of inputs to a model and observing changes in the output – analogous to classical linear regression, where we understand the concept of a change in the dependent variable for a unit-change in an independent variable.

To conclude this section, we define what we mean by **transparency** – using the same examples, both the neural network and the logistic regression models are equivalently **transparent** because the relationship between $\mathbf{x}$ and the data the network is "ingesting" is straight-forward; that is, there is no pre-processing or feature engineering/selection and we can assert that the activations of the input layer are identical to the data. We deem this to be an important property of the TIFU framework because if a model requires sophisticated pre-processing – for example, dimensionality reduction via principle components analysis with subsequent projection of each sample or input to the

[1] Of note, [3] do emphasise the concept of interpretability that we advocate for here

dimensionality-reduced feature space – clinicians will require further tools to understand how the data (representing patients in units of the original measurement scale) relates to the feature space the model operates on. It is not the case that pre-processing inputs to a model *precludes* transparency, rather, the engineering and presentation of the model must account for this transforming of inputs to a feature space so clinicians and patients can interrogate relationships.

Supplementary References

[1] A. Gelman, J. Hill, and A. Vehtari, *Regression and other stories.* Cambridge University Press, 2020.

[2] J. C. Gower, "Some distance properties of latent root and vector methods used in multivariate analysis," *Biometrika*, vol. 53, no. 3-4, pp. 325–338, 1966.

[3] M. T. Ribeiro, S. Singh, and C. Guestrin, "" why should i trust you?" explaining the predictions of any classifier," in *Proceedings of the 22nd ACM SIGKDD international conference on knowledge discovery and data mining*, pp. 1135–1144, 2016.

[4] S. Lipovetsky and M. Conklin, "Analysis of regression in game theory approach," *Applied Stochastic Models in Business and Industry*, vol. 17, no. 4, pp. 319–330, 2001.
